# Detection of MRSA in nasal swabs—marked reduction of time to report for negative reports by substituting classical manual workflow with total lab automation

**DOI:** 10.1007/s10096-018-3308-5

**Published:** 2018-06-25

**Authors:** Irene Burckhardt, Susanne Horner, Florian Burckhardt, Stefan Zimmermann

**Affiliations:** 1 Department for Infectious Diseases, Microbiology and Hygiene, University Hospital of Heidelberg, Im Neuenheimer Feld 324, 69115 Heidelberg, Germany; 2 Epiet Alumni Network, Heidelberg, Germany

**Keywords:** Automation, Time to result, MRSA, Workflow

## Abstract

In 2016, the workflow for MRSA detection in nasal swabs was changed from a classic-manual workflow to an automated workflow using total lab automation (TLA; BD Kiestra). This change entailed a reduction of the incubation time from 2 days to 20 h and reading of plates on weekdays and weekends instead of weekdays only. The workflow alteration did not include the introduction of 24/7. We wanted to follow up on the consequences for the times to report (TTR). We compared the TTR of all nasal swabs, which were sent for MRSA detection from June until August in 2015 (workflow—classic-manual) and in 2016 (workflow—automated). We calculated median TTR and interquartile ranges for the three possible reporting outcomes (negative, MRSA-known, MRSA-new) per day and workflow. A multivariable linear regression modeled the exposure variables workflow, day, and reporting outcome on TTR including interaction variables. The quantity and reasons for a TTR longer than 3 days were analyzed. During both 3-month periods, a total of 16,111 reports were issued (2015:7620; 2016:8491). The median TTR for negative reports was 48:28 (hh:mm) in 2015 and 23:58 in 2016. In the linear regression, all exposure variables had a strong and highly significant (*p* < 0.001) influence on the TTR. The number of reports with a TTR longer than 3 days shrank from 2418 (2015) to 60 (2016). The workflow alteration halved the median TTR for negative reports and the number of reports with a TTR longer than 3 days was reduced by 97.5%.

**Electronic supplementary material:**

The online version of this article (10.1007/s10096-018-3308-5) contains supplementary material, which is available to authorized users.

## Introduction

In recent years, total laboratory automation has been introduced in clinical microbiology laboratories, improving efficiency and decreasing time to results. Automation of specimen inoculation has reduced the need for manual processing and resulted in labor savings [1–4]. Standardization of incubation conditions (e.g., incubation atmosphere and temperature) and digital imaging of colony growth allows earlier processing of inoculated plates, potentially reducing time to final results. However, to fully take advantage of microbiology, automation requires the laboratory to alter their traditional workflow habits. For example, specimens should be processed when they are received in the laboratory rather than processing similar specimen types in batches.

One of the most commonly received specimens in our laboratory is nasal swabs for screening carriage of methicillin-resistant *Staphylococcus aureus* (MRSA).

Nasal carriage of MRSA is an important risk factor for infections with MRSA [5] as well as a risk factor for transmission to fellow patients and hospital staff [6]. Therefore, many societies for infectious diseases or comparable official bodies throughout Europe have issued screening recommendations for nasal carriage of MRSA [7]. Depending on guidelines, patients are either put into barrier isolation as soon as they are diagnosed with MRSA carriage or are kept in barrier isolation preemptively until carriage is excluded. In both cases, patients and staff benefit from a fast, accurate report. In the first case, an earlier diagnosis of MRSA carriage reduces the opportunity of transmission to others. In the second case, a fast negative result minimizes costly preemptive hygienic precautions. At our hospital, we follow a combined strategy depending on the history of the patient [8].

In 2016, we introduced automated workflow into our microbiology laboratory using BD Kiestra Total Lab Automation (TLA). Standardization of specimen processing and incubation conditions allowed us to modify our traditional workflow process of reading culture plates twice (after overnight incubation and after an additional 24 h) to reading the culture plates once (after 20 h of incubation). In this study, we report the impact of this workflow change on time to reported results.

## Materials and methods

### Patient samples and inoculation

The eSwab System (Copan) was used for all nasal swabs. In the classic workflow, we used the PREVI Isola system (BioMerieux, Nürtingen, Germany) for specimen inoculation and in the TLA workflow, we used the BD Kiestra TLA system. In both workflows, two plates were inoculated per patient material (10 μl each): a blood agar plate as growth control (Columbia agar 5% sheep blood, BD, Heidelberg, Germany) and an MRSA II agar plate (BD, Heidelberg, Germany) for specific MRSA detection.

We used the LIS Swisslab (RocheDiagnostics) for registration, documentation, and reporting of all samples.

### Working hours of the lab

Regular working hours at the microbiology lab at the Heidelberg University Hospital (reception desk) were 07:30 a.m.–05:30 p.m. on weekdays, 07:30 a.m.–12:00 p.m. on Saturdays, and 08:30 a.m.–12:00 p.m. on Sundays and public holidays. All samples arriving during these times were registered and inoculated. Incubation started the same day they were received. Working hours and sample processing times did not change during the study periods.

### Classic workflow

After registration of samples (day 0), swabs were placed in specialized PREVI Isola racks and plates were streaked immediately. Inoculated plates were immediately transported to incubators and incubated at 35 °C until reading. The delay between inoculation of plates and placement in the incubator was not recorded. First, reading was performed the next weekday (day 1). Reading normally started at 08:00 a.m. and was finished by 01:00 p.m. The second and final reading was done the next weekday (day 2). Incubation times at first and second reading can only be roughly estimated: read 1, 15 h–24 h and read 2, 39 h–48 h (weekdays). There was no reading on Saturdays, Sundays, or public holidays.

### TLA workflow

After registration of samples (day 0), swabs were put into the specialized TLA racks and were streaked immediately (streaking pattern 5) and automatically transported to the incubators. Plates were incubated at 35 °C until scheduled imaging in the camera unit. Imaging time was 20 h after start of incubation. Twenty hours was chosen because a previous validation had shown that 99% of all MRSA were detected after 20 h of incubation (Burckhardt et al., submitted). Reading of plate images was done as soon as possible after imaging. On weekdays, reading normally started at 08:00 a.m. and was finished by 03:30 p.m. when all plates from the previous day were read. In the TLA workflow, reading started at 10:00 a.m. on Saturdays, Sundays, and public holidays and ended at 12:00 p.m.

### Reading and reporting

Reading of MRSA plates had three reporting outcomes independent of workflow: (A) no growth on MRSA agar (“negative”), (B) growth of mauve colonies indicative of MRSA and patient already known as MRSA carrier (“MRSA-known”), and (C) growth of mauve colonies indicative of MRSA and patient not yet known as MRSA carrier (“MRSA-new”).

In case (C), we performed a confirmatory PCR (*mecA*) for MRSA on one of the mauve colonies. The reason for this confirmation was that false-positive growth appeared on the MRSA II agar typically on day 2 during classic reading and the enormous consequences for therapy and hospital hygiene in case of positivity [9]. PCR runs were available only at 01:00 p.m. on weekdays for both workflows. These samples were only considered positive for MRSA if the PCR was positive.

Specimens without any growth on MRSA agar and on the control blood agar plate during the final read were reported as “no screening report possible.” For statistical analysis, these samples were included into the reporting outcome as “negative” because these samples had the same runtime as the negative reports. They were neither specified nor analyzed separately except in Table S1 (see Online Resource Table S1).

In the classic workflow, reports were generated as soon as a sample was positive for MRSA or negative on day 2. In the TLA workflow, a report was generated as soon as a sample was positive for MRSA or negative after reading the 20-h image.

### Data analysis

We compared the times to report (TTR) for all nasal swabs sent for MRSA detection during the following 3-month time periods: classic workflow—June 1 to August 31, 2015; TLA workflow—June 1 to August 31, 2016. In this paper, we defined the TTR as the time period between registration of a patient sample in our LIS and the electronic dispatch of the final report to the respective ward. The procedures for registration and reporting were not changed during the study periods. We rounded the results to the full minute. The TTR is depicted as hh:mm throughout this paper.

Data were analyzed using Stata Statistical Software Release 12. Descriptive data analyses included the calculation of median TTR, and description of maximum and minimum TTR. Data were stratified into workflow (represented by year: 2015—classical manual workflow and 2016—automated workflow using a TLA), day (Monday to Sunday), and reporting outcome (negative, MRSA-known, and MRSA-new). Boxplots for TTR per day were created for each workflow, specifying the median, interquartile range, maximum, minimum, and outliers for negative, MRSA-known, and MRSA-new reports. To quantify the effect of the workflow changes on the TTR, we calculated a multivariable linear regression. We modeled the influence of workflow, day, reporting outcome (exposure variables), and the interaction of workflow with day and the interaction of workflow with reporting outcome on the dependent variable TTR. Interaction variables were included because the additional reading on weekends in 2016 reduced the TTR for Thursday, Friday, and Saturday samples only. Similarly, the interaction between workflow and reporting outcome was necessary because positive TLA result that were read after 01.00 p.m. in the automated workflow did not make it into that days PCR run. In the manual workflow, all plates were read until 01.00 p.m. and all “MRSA-new” samples were included in the PCR run the same day. The outlier analysis of samples with a TTR ≥ 3 days was done by reviewing the respective reports in the LIS.

## Results

During the classic workflow period, we received 7747 nasal swabs of which 7620 could be inoculated (see Online Resource Table S1). The main reason for non-inoculation was an empty sample tube. A total of 323 specimens showed no growth on the control blood agar, 7198 specimens were negative, and 99 specimens (1.3%) were positive. During the TLA workflow period, we received 8593 nasal swabs (10.9% increased volume) of which 8491 could be inoculated. A total of 326 samples showed no growth on the blood agar, 8061 specimens were negative, and 104 specimens (1.2%) were positive.

**Fig. 1:**
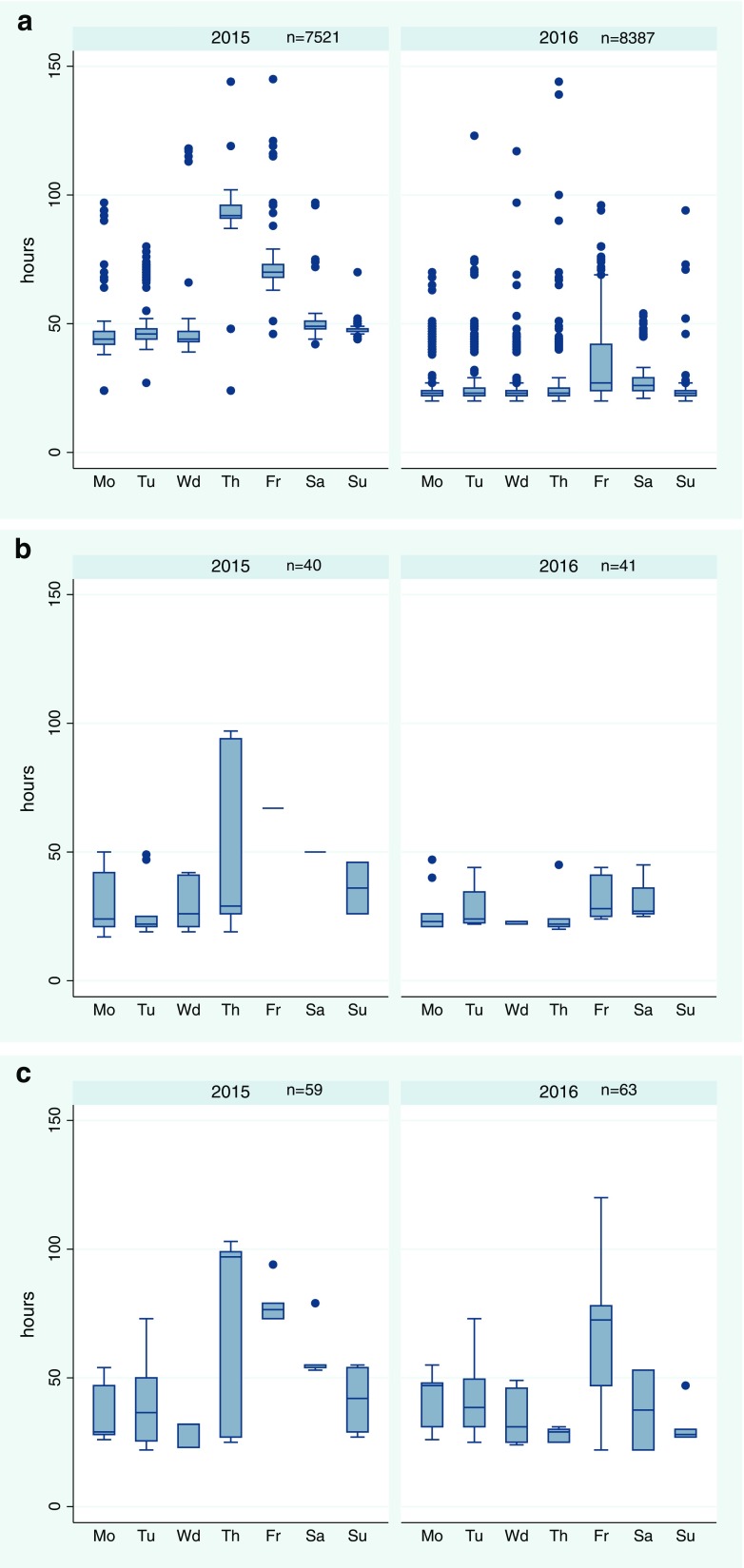
TTR of 7620 (2015) and 8491 (2016) samples stratified according to workflow, day, and result (**a** negative, **b** MRSA-known, **c** MRSA-new) depicted are median, interquartile range (IQR), and outliers; whiskers are defined as 1.5× IQR; Mo, Monday; Tu, Tuesday; Wd, Wednesday; Th, Thursday; Fr, Friday; Sa, Saturday; Su, Sunday

The median TTR for *negative reports* was 48:28 (hh:mm) in 2015 and 23:58 (hh:mm) in 2016 (see Fig. 1 and Online Resource Table S2). Depending on the day of arrival, the median TTR for negative reports varied between 44:29 (Monday) and 92:45 (Thursday) in 2015, and 23:08 (Monday) and 27:37 (Friday) in 2016.

The median TTR for *positive reports* for known MRSA carriers was 26:33 in 2015 and 24:39 in 2016. Depending on the day of arrival, samples arriving on Tuesday in 2015 had the shortest median TTR (22:21) and samples arriving on Friday had the longest median TTR (67:11). The corresponding shortest median TTR in 2016 was Thursday (22:43) and the longest was Friday (28:15).

The median TTR for *positive reports* for patients newly diagnosed with MRSA was 50:24 and 31:40 for 2015 and 2016, respectively. It varied between 29:35 (Monday) and 97:16 (Thursday) in 2015 and 28:23 (Sunday) and 72:57 (Friday) in 2016.

All variables included into the multivariable linear regression analysis were highly significant with *p* < 0.001. The TTR for a Monday sample with a negative report in 2015 served as the baseline for interpretation. In general, the TTR for 2016 samples was 20:01 shorter. Compared to the baseline, the TTR for non-Monday samples was longer between 1:20 (Wednesday samples) and 47:53 (Thursday samples). Positive reports for samples of patients already known to carry MRSA were 19:39 faster and positive reports for newly diagnosed MRSA carriers were 7:31 faster than the baseline (see Table 1).

**Table 1:** Multivariable linear regression

Independent variables and interactions	TTR difference (hh:mm)	95% CI
Workflow (2016, automated)	− 20:01*	[− 20:30; − 19:30]
Day (Tuesday)	2:53*	[2:24; 3:24]
Day (Wednesday)	1:20*	[0:48; 1:54]
Day (Thursday)	47:53*	[47:18; 48:24]
Day (Friday)	26:01*	[25:30; 26:36]
Day (Saturday)	5:39*	[4:54; 6:24]
Day (Sunday)	3:15*	[2:24; 4:06]
Reporting outcome (MRSA-known)	− 19:39*	[− 21:54; − 17:24]
Reporting outcome (MRSA-new)	− 7:31*	[− 9:24; − 5:36]
Workflow (2016, automated) × day (Tuesday)	− 1:18*	[− 2:00; − 0:36]
Workflow (2016, automated) × day (Wednesday)	− 1:41*	[− 2:24; − 0:54]
Workflow (2016, automated) × day (Thursday)	− 48:09*	[− 48:54; − 47:24]
Workflow (2016, automated) × day (Friday)	− 18:52*	[− 19:36; − 18:06]
Workflow (2016, automated) × day (Saturday)	− 2:49*	[− 3:54; − 1:48]
Workflow (2016, automated) × day (Sunday)	− 4:09*	[− 5:18; − 3:00]
Workflow (2016, automated) × reporting outcome (MRSA-known)	20:32*	[17:24; 23:42]
Workflow (2016, automated) × reporting outcome (MRSA-new)	22:40*	[20:06; 25:18]
Constant	45:05	[44:42; 45:24]

95% confidence intervals (CI) in brackets; reference sample: workflow (2015), day (Monday), reporting outcome (negative); *p < 0.001; dependent variable, time to report (TTR); total observations: 16,111

The interaction between workflow and day showed a reduction of the TTR particularly for Thursday samples (48:09) but also for Friday samples (18:52). TTR reductions on the other days were moderate (1:18–4:09). The interaction between workflow and reporting outcome showed that the new automated workflow accelerated MRSA-known reports only by 2 h compared to MRSA-new reports (20:32 vs 22:40).

To evaluate the effect of the workflow changes on outliers, we reviewed all samples with reports issued on day 3 or later. During the 3 months in 2015, 2418 of 7620 (31.7%) reports were issued on day 3 or later. One thousand one hundred eight-two samples were Friday samples, 1077 were Thursday samples, and 112 samples were sent 2 days before a public holiday. For the remaining 47 samples, the analysis revealed that in 17 cases, the MRSA strain grew suboptimal and had to be subcultured, in 16 cases, the sample had to be inoculated again and in 14 cases, we could not determine why the report was issued that late. In 2016, only 60 of 8491 (0.71%) reports were issued on day 3 or later. In 3 cases, the MRSA strain grew suboptimal and had to be subcultured and in 9 cases, the sample had to be inoculated again. In 48 cases, we could not identify the reason for the late report (see Table 2).

**Table 2:** Analysis of samples with TTR ≥ 3 days

	*N*	% of all samples per study period*
2015	*2418*	*31.72*
Sample arrived on a Friday	1182	15.51
Sample arrived on a Thursday	1077	14.13
Sample arrived on a public holiday	112	1.47
Slow growth^†^	17	0.22
Repeat inoculation^‡^	16	0.21
Unclear^§^	14	0.18
2016	*60*	*0.72*
Unclear	48	0.57
Repeat inoculation	9	0.11
Slow growth	3	0.04
Total	*2478*	*32.44*

*% of all samples inoculated: 7620 (2015) and 8491 (2016)

^†^Slow growth—MRSA strains grew very tiny and had to be subcultured before further work-up

^‡^Repeat inoculation—samples had to be inoculated again mainly because the plates could not be found or were not suitable for reading

^§^Unclear—review of LIS data did not reveal a documented explanation for the long TTR

## Discussion

A quick, accurate report is what infection control specialist and clinicians expect from their microbiology laboratory. The earlier a report arrives, the faster an evidence-based therapy or hygienic precautions can be implemented or withdrawn. In our opinion, two main factors influence the speed of microbiology reports: (A) the time it takes for bacteria to grow on agar media and (B) the workflow in the laboratory. We addressed the second factor by implementing a TLA (BD Kiestra Total Lab Automation) and by extending reading times for plates to weekends and public holidays.

Until 14th January 2016, the workflow for nasal swabs for screening for MRSA consisted of inoculation of all samples 7 days a week and reading of plates on weekdays. Due to the fact that inoculation times for individual plates were not recorded, we read these samples on two consecutive days (day 1 and 2), except if samples were already positive on day 1. In case day 1 and/or 2 fell on a weekend or public holiday, reading was postponed to the next weekday. This had been a concession to the availability of staff during weekends and public holidays. With the implementation and go-live of the TLA on the 15th January 2016, the incubation time for MRSA plates could be reduced to 20 h and plates were read 7 days a week.

As a consequence, on average, the median TTR for negative reports went down from 48:28 (2015) to 23:58 (2016), which is less than half. The median TTR for Thursday samples even fell to a third (92:45 (2015)–23:18 (2016)). Reason was that in the classical system, the second read was postponed until Monday whereas in the automated workflow with a single image after 20 h, the reports could be issued on Friday already. Therefore, this enormous shortening of the median TTR is directly attributable to the introduction of the TLA and the shortening of the incubation time of the agar plates. The median TTR for Friday samples fell from 70:43 (2015) to 27:37 (2016) and for Saturday samples from 49:55 (2015) to 26:06 (2016). Samples from both days benefited from a combination of reduction of incubation time and reading of plates during weekends.

The median TTR for samples from patients with known MRSA colonization (MRSA-known) did not change substantially (26:33 (2015), 24:39 (2016)). However, it must be noted that although all TLA culture plates were examined after 20 h of incubation, the classic-manual plates were initially examined after incubation for 15 to 24 h (dependent on the time the specimen was processed the previous day). For this reason, a dramatic reduction in TTR with positive specimens would not be expected unless a significant number of positive specimens were only detected on day 2. In fact, 60% of all MRSA-known specimens were identified on day 1 in the classic workflow. The median TTR for positive reports (MRSA-new) was reduced from 50:24 (2015) to 31:40 (2016). This is due to the fact that only 46% of the MRSA-new specimens were identified on day 1.

The general difference in both workflows between MRSA-known and MRSA-new was the fact that for MRSA-new, a PCR was done to confirm the presence of *mecA*. Therefore, the increased median TTR for MRSA-new (compared to MRSA-known) in 2016 of about 7 h must be an effect of the availability of the PCR. It is done only on weekdays at 01.00 p.m. It is generally not available on weekends and public holidays. A median TTR for Friday samples (MRSA-new) of 72:57 even with the automated workflow is a direct consequence of the non-availability of the PCR during weekends.

The advantage of a linear regression over a mere descriptive analysis of TTR (median, interquartile range, minimum, maximum) is that it quantifies the influence of the respective exposure variables. The analysis allows three conclusions. (A) The workflow change from classic-manual (2015) to automated-TLA (2016) reduced the TTR by 20:01, i.e., it almost halved the median TTR. (B) The introduction of the TLA and introduction of reading 7 days a week minimized the effect of the day of arrival on the TTR. With the exception of Friday and Saturday samples, the effect of day on the TTR is not bigger than 2:00. The reason for the bigger difference for Friday and Saturday samples is that these samples are read on Saturday and Sunday. Even in the new workflow, reading ends at 12:00 p.m. on these days in contrast to 03:30 p.m. on weekdays. (C) The workflow change did not alter the TTR for positive reports (MRSA-known). This is explained by the fact that in the classic workflow, the majority of positive reports for known MRSA carriers were issued on day 1.

Of note, the aspect of the MRSA colonies did not change with incubating the plates in the TLA incubators instead of using classic stand-alone incubators. The morphology of the colonies was identical during the two study periods.

In principle, the reduction of the incubation time of the MRSA chromogenic agar could be done in a non-automated workflow, too. However, documenting the inoculation times of hundreds of plates each day is laborious and error prone. In our opinion, using an automated system is the most convenient and reliable solution.

Our study has several limitations. First, we did not look at earlier imaging/reading time points than 20 h. It would have meant to read 8491 samples twice to potentially reduce the TTR of 63 samples (0.74%; 2016; MRSA-new). This amount of additional work would not have been feasible for the technicians. Second, we changed two important workflow parameters at a single time point. This complicated the attribution of time gains. However, modeling a multivariable linear regression accounted for that. The inclusion of the variable “day” into the analysis enabled us to disentangle the influence of the implementation of the TLA from the fact that reading is now done on 7 days per week. Third, we did not collect any data on the length of barrier isolation times on the wards. Because the main benefit in terms of time to report is for negative reports, we expect that the length of preemptive isolation decreased by 1 day. However, this effect is not only dependent on the time to report of the microbiology laboratory but also on the reaction time of the clinicians to the report, i.e., the workflow on the wards.

## Conclusion

The combined workflow alteration of implementation of a TLA and reading plates on weekdays and weekends halved the median TTR for negative reports (> 98.5% of all reports in 2016). The reduction of the TTR for Monday to Thursday samples is due to automation and the reduction of incubation time. However, as soon as Saturday, Sunday, and public holidays fall into the time sequence of reading, the effects are due to both changes. In addition, the number of reports, which had a TTR of 3 days or longer, was reduced by 97.5%. A switch to working 24/7 might further reduce TTR and avoid TTRs of longer than 3 days.

## Electronic supplementary material

Table S1 (DOCX 16 kb)

Table S2 (DOCX 19 kb)

## Abbreviations

TLA: Total lab automation
TTR: Time to report
