# Supplementary material for: Detection of MRSA in nasal swabs—marked reduction of time to report for negative reports by substituting classical manual workflow with total lab automation
Source: Eur J Clin Microbiol Infect Dis. 2018 Jun 25;37(9):1745–51. doi: 10.1007/s10096-018-3308-5 (PMC6133036; doi:10.1007/s10096-018-3308-5)
Supplement: Supplementary file 1 — (DOCX 16 kb) [file 10096_2018_3308_MOESM1_ESM.docx]

|  | **June 2015** | **July 2015** | **August 15** | **Sum 2015** |  | **June 2016** | **July 2016** | **August 16** | **Sum 2016** |  | **Total** |
| --- | --- | --- | --- | --- | --- | --- | --- | --- | --- | --- | --- |
| **all nasal swabs** | 2,606 | 2,678 | 2,463 | **7,747** |  | 2,534 | 2,959 | 3,100 | **8,593** |  | **16,340** |
| **swabs inoculated** | 2,563 | 2,637 | 2,420 | **7,620** |  | 2,507 | 2,910 | 3,074 | **8,491** |  | **16,111** |
| **no growth on control plate** | 118 | 114 | 91 | **323** |  | 124 | 85 | 117 | **326** |  | **649** |
| **negative** | 2,411 | 2,491 | 2,296 | **7,198** |  | 2,357 | 2,786 | 2,918 | **8,061** |  | **15,259** |
| **MRSA positive** | 34 | 32 | 33 | **99** |  | 26 | 39 | 39 | **104** |  | **203** |
|  |  |  |  |  |  |  |  |  |  |  |  |

**Table S1**: Samples received during the study periods in 2015 and 2016 per month
