# Supplementary material for: Detection of MRSA in nasal swabs—marked reduction of time to report for negative reports by substituting classical manual workflow with total lab automation
Source: Eur J Clin Microbiol Infect Dis. 2018 Jun 25;37(9):1745–51. doi: 10.1007/s10096-018-3308-5 (PMC6133036; doi:10.1007/s10096-018-3308-5)
Supplement: Supplementary file 2 — (DOCX 19 kb) [file 10096_2018_3308_MOESM2_ESM.docx]

|  | **2015 – classical manual** | | | | | | | | | | | | | |
| --- | --- | --- | --- | --- | --- | --- | --- | --- | --- | --- | --- | --- | --- | --- |
|  | **negative** | | | | **positive (MRSA-known)** | | | | | **positive (MRSA-new)** | | | | |
|  | N | median | min | max |  | N | median | min | max |  | N | median | min | max |
| **Monday** | 1,491 | 44:29 | 24:23 | 97:08 |  | 13 | 24:37 | 17:59 | 50:36 |  | 13 | 29:35 | 26:25 | 54:38 |
| **Tuesday** | 1,516 | 46:02 | 27:13 | 80:22 |  | 9 | 22:21 | 19:40 | 49:13 |  | 12 | 37:24 | 22:51 | 73:50 |
| **Wednesday** | 1,362 | 44:51 | 39:39 | 118:42 |  | 7 | 26:58 | 19:41 | 42:53 |  | 3 | 32:06 | 23:55 | 32:51 |
| **Thursday** | 1,133 | 92:45 | 24:39 | 144:26 |  | 7 | 29:38 | 19:18 | 97:16 |  | 11 | 97:16 | 25:43 | 103:13 |
| **Friday** | 1,238 | 70:43 | 46:14 | 145:18 |  | 1 | 67:11 | 67:11 | 67:11 |  | 6 | 77:03 | 73:32 | 94:36 |
| **Saturday** | 458 | 49:55 | 42:48 | 97:59 |  | 1 | 50:10 | 50:10 | 50:10 |  | 6 | 55:24 | 53:39 | 79:47 |
| **Sunday** | 323 | 48:06 | 44:01 | 70:30 |  | 2 | 36:49 | 26:52 | 46:47 |  | 8 | 42:25 | 27:16 | 55:01 |
| **Total** | 7,521 | 48:28 | 24:23 | 145:18 |  | 40 | 26:33 | 17:59 | 97:16 |  | 59 | 50:24 | 22:51 | 103:13 |
|  |  |  |  |  |  |  |  |  |  |  |  |  |  |  |
|  | **2016 - automated** | | | | | | | | | | | | | |
|  | **negative** | | | | **positive (MRSA-known)** | | | | | **positive (MRSA-new)** | | | | |
|  | N | median | min | max |  | N | median | min | max |  | N | median | min | max |
| **Monday** | 1,494 | 23:08 | 20:37 | 70:44 |  | 11 | 23:31 | 21:19 | 47:51 |  | 15 | 47:52 | 26:02 | 55:16 |
| **Tuesday** | 1,622 | 23:54 | 20:29 | 123:04 |  | 4 | 24:21 | 22:44 | 44:05 |  | 12 | 39:01 | 25:03 | 73:43 |
| **Wednesday** | 1,510 | 23:30 | 20:29 | 117:29 |  | 5 | 23:42 | 22:33 | 23:49 |  | 10 | 31:39 | 24:51 | 49:22 |
| **Thursday** | 1,446 | 23:18 | 20:04 | 144:42 |  | 6 | 22:43 | 20:59 | 45:15 |  | 9 | 29:03 | 25:21 | 31:24 |
| **Friday** | 1,364 | 27:37 | 20:56 | 96:11 |  | 11 | 28:15 | 24:06 | 44:51 |  | 8 | 72:57 | 22:39 | 120:44 |
| **Saturday** | 532 | 26:06 | 21:06 | 54:49 |  | 4 | 27:40 | 25:33 | 45:11 |  | 2 | 37:52 | 22:02 | 53:42 |
| **Sunday** | 419 | 23:22 | 20:50 | 94:17 |  | 0 | 0:00 | 0:00 | 0:00 |  | 7 | 28:23 | 27:48 | 47:38 |
| **Total** | 8,387 | 23:58 | 20:04 | 144:42 |  | 41 | 24:39 | 20:59 | 47:51 |  | 63 | 31:40 | 22:02 | 120:44 |

**Table S2**: samples stratified according to day, workflow and reporting outcome; median, minimum (min) and maximum (max) TTR are shown (hh:mm).
